# Chronic Kidney Disease Is Positively and Diabetes Mellitus Is Negatively Associated with Abdominal Aortic Aneurysm

**DOI:** 10.1371/journal.pone.0164015

**Published:** 2016-10-20

**Authors:** Hidemi Takeuchi, Michihiro Okuyama, Haruhito A. Uchida, Yuki Kakio, Ryoko Umebayashi, Yuka Okuyama, Yasuhiro Fujii, Susumu Ozawa, Masashi Yoshida, Yu Oshima, Shunji Sano, Jun Wada

**Affiliations:** 1 Department of Nephrology, Rheumatology, Endocrinology and Metabolism, Okayama University Graduate School of Medicine, Dentistry and Pharmaceutical Sciences, Okayama, Japan; 2 Department of Cardiovascular Surgery, Okayama University Hospital, Okayama, Japan; 3 Department of Chronic Kidney Disease and Cardiovascular Disease, Okayama University Graduate School of Medicine, Dentistry and Pharmaceutical Sciences, Okayama, Japan; 4 Department of Cardiovascular Surgery, Kure Kyosai Hospital, Hiroshima, Japan; Universita degli Studi di Perugia, ITALY

## Abstract

**Background and Aims:**

Chronic kidney disease (CKD) and diabetes mellitus (DM) are considered as risk factors for cardiovascular diseases. The purpose of this study was to clarify the relationship of CKD and DM with the presence of abdominal aortic aneurysm (AAA).

**Methods:**

We enrolled 261 patients with AAA (AAA+) and age-and-sex matched 261 patients without AAA (AAA-) at two hospitals between 2008 and 2014, and examined the association between the risk factors and the presence of AAA. Furthermore, in order to investigate the prevalence of AAA in each group, we enrolled 1126 patients with CKD and 400 patients with DM.

**Results:**

The presence of CKD in patients with AAA+ was significantly higher than that in patients with AAA- (AAA+; 65%, AAA-; 52%, *P* = 0.004). The presence of DM in patients with AAA+ was significantly lower than that in patients with AAA- (AAA+; 17%, AAA-; 35%, *P* < 0.001). A multivariate logistic regression analysis demonstrated that hypertension, ischemic heart disease and CKD were independent determinants, whereas, DM was a negatively independent determinant, for the presence of AAA. The prevalence of AAA in patients with CKD 65 years old and above was 5.1%, whereas, that in patients with DM 65 years old and above was only 0.6%.

**Conclusion:**

CKD is a positively associated with the presence of AAA. In contrast, DM is a negatively associated with the presence of AAA in Japanese population.

## Introduction

Abdominal aortic aneurysm (AAA) is the most popular aortic aneurysm. Previous hospital- and population-based studies reported that the estimated prevalence of AAA in developed countries is 4 to 9% [1–6]. Most of AAAs are asymptomatic until rupture. However, once rupture occurs, it mostly leads to a rapid clinical course and results in sudden death. Mortality rates are as high as 90%, and 50 to 70% cases who were taken in the operating room died [7–9]. The incidence of AAA increases with age, particularly over 60 years old [3, 5, 10]. Since it is expected that the number of elderly population increases in future, the prevalence of AAA could increase substantially.

In general, the risk factors for AAA are smoking, male gender, aging, Caucasian race, hypertension and family history of AAA [10–12]. These risk factors often overlap with many of the classical risk factors for atherosclerosis. Recently, chronic kidney disease (CKD) [13] has been recognized as one of the risk factors that promotes atherosclerosis as well as cardiovascular disease (CVD). Both the decline of glomerular filtration rate (GFR) and the increase of urinary protein excretion are independent risk factors for CVD [13–16]. However, the association between CKD and AAA remains unknown. Diabetes Mellitus (DM) is also considered to be one of the major risk factors for atherosclerosis. However, DM has been reported to exert favorable effects on the incidence and development of AAA [4, 17–20]. Indeed, a few studies raised the possible pathophysiological mechanisms of the relationship between DM and AAA, that is, the cross-interaction among the extracellular matrix, inflammatory cells, the chronic glucose elevation and advanced glycated end products (AGEs) [21–26]. Since these studies were conducted in the western population, such a protective role of DM on AAA was not explored in the Asian population.

The purpose of this study was to clarify the impact of CKD and DM on the presence of AAA. We performed a cross-sectional retrospective case-control study in Japanese population.

## Materials and Methods

### Study participants

To examine the relationship of cardiovascular risk factors with the presence of AAA, we retrospectively enrolled 261 patients who were diagnosed with AAA by abdominal computed tomographic scanning (CT) at the department of Cardiovascular Surgery of Okayama University hospital and Kure Kyosai hospital between January 2008 and December 2014 as the AAA+ group. We also enrolled age-and-sex matched 261 patients from more than ten-thousand patients who received abdominal CT except at the department of Cardiovascular Surgery as the control (AAA-) group during same period. Furthermore, in order to investigate the prevalence of AAA, we retrospectively enrolled 1126 patients who underwent abdominal CT at the department of Nephrology as the CKD group and 400 patients who underwent abdominal CT at the department of Diabetes as the DM group.

### Evaluation items and criteria

AAA was defined as the maximum abdominal aortic diameter in minor axis ≥ 3.0 cm on CT in our study [3]. By checking medical records, cardiovascular risk factors of each patient were evaluated: body mass index (BMI), hypertension (HTN), dyslipidemia (DLP), DM, CKD, smoking habit, ischemic heart disease (IHD) and stroke. HTN was defined as an office blood pressure of 140/90 mmHg and above. The patients who took antihypertensive agent were also considered to have HTN. DLP was defined as the serum concentration of low-density lipoprotein cholesterol of 140 mg/dL and above, the serum concentration of high-density lipoprotein cholesterol of below 40 mg/dL, or the serum concentration of triglyceride of 150 mg/dL and above. The patients who took anti-lipidemic agent were also considered to have DLP. DM was defined as the level of HbA1c (NGSP) of 6.5% and above. The patients who took anti-diabetic agents were regarded to have DM. CKD was defined as the level of estimated GFR (eGFR) [27] of below 60 mL/min/1.73m^2^ and/or proteinuria [13]. Proteinuria was defined to be positive in the urine dip stick examination. Smoking habit were defined as a current or former smoking habit. IHD was defined as a symptomatic angina pectoris or myocardial infarction that required the treatment of percutaneous coronary artery intervention or coronary artery bypass grafting. Stroke was defined as a symptomatic cerebral infarction or hemorrhage.

### Ethics

This study followed the Declaration of Helsinki (seventh revision, 2013) on medical protocol and ethics. This is a retrospective observational study. We corrected the data only from physician’s chart, therefore our Institutional Review Board waived the requirement of written informed consent but requested to give the opportunity to the patients to reject the enrollment of this study by leaflets or website of our hospital. Finally, the ethics committees of Okayama University Institutional Review Board (accredited ISO9001/2000), Okayama, Japan and Kure Kyosai hospital Institutional Review Board approved the protocol.

### Statistics

All data were expressed as the mean ± standard deviation. Differences between AAA+ group and AAA- group were examined by unpaired t-test or chi-square test. Odds ratio were studied to evaluate the risk factor for AAA. Multiple logistic regression analysis was carried out to examine the independent associations among risk factors. A difference of *P* < 0.05 was taken as statistically significant. All the data were analyzed using Sigma Stat for Windows (version 3.5, Systat Software Inc., San Jose, California, USA).

## Results

### Characteristics of Participants

The characteristics of participants were shown in Table 1. The participants of AAA+ group included 203 of male and 58 of female. Average age of AAA+ group was 77.0 ± 8.3 years old. The number of subjects, sex ratio and age of AAA- group completely matched AAA+ group. There were no differences between two groups in BMI and serum creatinine concentration. eGFR in AAA+ group was significantly lower than that in AAA- group (AAA+; 54.4 ± 21.2 mL/min/1.73m^2^, AAA-; 61.4 ± 26.2 mL/min/1.73m^2^, *P* < 0.001), despite matched age and sex ratio. However, the level of HbA1c in AAA- group was significantly higher than that in AAA+ group (AAA+; 5.7 ± 0.6%, AAA-; 5.9 ± 0.9%, *P* = 0.034).

**Table 1:** Clinical Characteristics of Patients With and Without AAA.

	AAA+ (n = 261)	AAA- (n = 261)	P value
Age, years	77.0 ± 8.3	77.0 ± 8.3	
Sex (male), n (%)	203 (78%)	203 (78%)	
Height, (cm)	160.6 ± 8.8	159.5 ± 8.4	0.184
Weight, (kg)	58.6 ± 11.8	56.3 ± 11.1	0.025*
Body mass index, (kg/m^2^)	22.7 ± 3.8	22.1 ± 3.8	0.140
Cr, (mg/dl)	1.28 ± 1.19	1.18 ± 1.27	0.390
eGFR, (ml/min/1.73m^2^)	54.4 ± 21.2	61.4 ± 26.2	< 0.001**
HbA1c, (%)	5.7 ± 0.6	5.9 ± 0.9	0.034*
Ant-Hypertensive therapy			
Angiotensin-receptor blocker	36.2%	34.5%	
Angiotensin-converting enzyme inhibitor	20.7%	2.6%	
Calcium-channel blocker	41.4%	34.5%	
Diuretic	18.1%	8.6%	
β-blocker	25.0%	8.6%	
Diabetic therapy			
Insulin	2.6%	7.8%	
Pioglitazone	4.3%	2.6%	
Metformin	0%	2.6%	
Sulfonylurea	2.6%	6.9%	
DPP4-Inhibitor	3.4%	7.8%	
Others			
Statin	37.9%	17.2%	
EPA	0.9%	2.6%	
Anti-Platelet therapy	32.8%	15.5%	

Data are presented as mean value ± standard deviation or n (%) of patients. AAA, abdominal aortic aneurysm; Cr, creatinine; eGFR, estimated glomerular filtration rate; Hb, Hemoglobin; DPP, Dipeptidyl Peptidase; EPA, eicosapentaenoic acid. P values are obtained by unpaired student’s t-test.

*P < 0.05

**P < 0.01

### Presence of risk factors for AAA

As shown in Table 2, the presence of the risk factors in AAA+ group, such as HTN, DLP and CKD, were significantly higher than that in AAA- group. Whereas, the presence of DM in AAA+ group was significantly lower than in AAA- group (AAA+; 17%, AAA-; 35%, *P* < 0.001). No differences in smoking habit and the history of stroke were observed between groups. The presence of IHD in AAA+ group was 21% higher than in AAA- group (*P* < 0.001).

**Table 2:** Prevalence of Risk Factor and Past History.

	AAA+ (n = 261)	AAA- (n = 261)	P value
CKD, n (%)	170 (65%)	136 (52%)	0.004**
DM, n (%)	44 (17%)	91 (35%)	< 0.001**
HTN, n (%)	202 (77%)	160 (61%)	< 0.001**
DLP, n (%)	132 (51%)	103 (39%)	0.017*
Smoking habit (Current + Former), n (%)	152 (58%)	130 (50%)	0.071
Current smoker, n (%)	44 (17%)	37 (14%)	
Former smoker, n (%)	108 (41%)	93 (36%)	
Never smoked, n (%)	96 (37%)	116 (44%)	
History of IHD, n (%)	85 (33%)	31 (12%)	< 0.001**
History of stroke, n (%)	31 (12%)	39 (15%)	0.397

Data are presented as n (%) of patients. AAA, abdominal aortic aneurysm; CKD, chronic kidney disease; DM, diabetes mellitus; HTN, hypertension; DLP, dyslipidemia; IHD, ischemic heart disease. P values are obtained by chi-square test.

*P < 0.05

**P < 0.01

### Regression analysis

Results of univariate and multivariate logistic regression analysis of factors associated with AAA were shown in Table 3. HTN, DLP, CKD, smoking habit and the history of IHD were positively associated with AAA by a univariate regression analysis. Only DM was negatively associated with AAA (OR; 0.381, 95%C.I.; 0.252–0.575, *P* < 0.001). The history of stroke was not significantly correlated with the presence of AAA. In addition, a multivariate logistic regression analysis revealed that HTN, CKD and the history of IHD were independent determinants, whereas, DM was the only independent negative determinant, for the presence of AAA.

**Table 3:** Univariate and Multivariate Analysis for the Presence of AAA.

	Univariate analysis			Multivariate analysis model 1			Multivariate analysis model 2		
	Odds ratio	95% C.I.	P value	Odds ratio	95% C.I.	P value	Odds ratio	95% C.I.	P value
CKD	1.710	1.197–2.443	0.003**	1.606	1.068–2.417	0.023*	1.603	1.064–2.414	0.024*
DM	0.381	0.252–0.575	< 0.001**	0.306	0.191–0.489	< 0.001**	0.301	0.188–0.482	< 0.001**
HTN	2.237	1.552–3.289	< 0.001**	1.880	1.224–2.889	0.004**	1.874	1.219–2.881	0.004**
DLP	1.554	1.097–2.202	0.013*	1.374	0.920–2.052	0.120	1.367	0.915–2.043	0.127
Smoking habit	1.439	1.015–2.040	0.041*	1.518	0.982–2.347	0.060	1.476	0.951–2.288	0.082
History of IHD	3.683	2.332–5.816	< 0.001**	3.754	2.255–6.250	< 0.001**	3.819	2.289–6.374	< 0.001**
History of Stroke	0.777	0.468–1.290	0.330				0.708	0.400–1.254	0.236

AAA, abdominal aortic aneurysm; CKD, chronic kidney disease; DM, diabetes mellitus; HTN, hypertension; DLP, dyslipidemia; IHD, ischemic heart disease; C.I., confidence interval. In Multivariate analysis model 1, adjusted for items that significant difference was out with univariate analysis, age and sex. In Multivariate analysis model 2, adjusted for all items, age and sex. P values are obtained by multiple logistic regression analysis.

*P < 0.05

**P < 0.01

### Prevalence of AAA in patients with CKD or DM

We next investigated the prevalence of AAA in patients with CKD or DM. Firstly, total 1126 patients (male / female: 612 / 524) with CKD were examined. Average age of CKD group was 56.4 ± 19.0 years old. The number of patients 55 years old and above, 65 years old and above, and 75 years old and above were 670 (male / female: 380 / 290), 468 (male / female: 277 / 191) and 207 (male / female: 116 / 91), respectively (Table 4). The prevalence of AAA in the patients with CKD was 2.5% in all patients, 3.9% in patients 55 years old and above, 5.1% in patients 65 years old and above and 7.2% in patients 75 years old and above, respectively.

**Table 4:** Prevalence of AAA in CKD group or DM group.

	CKD group (n = 1126)	DM group (n = 400)
Age, years	56.4 ± 19.0	58.5 ± 15.9
Sex (male), n (%)	612 (54.4%)	202 (50.5%)
55 y.o. and above, n (%)	670 (59.5%)	271 (67.8%)
65 y.o. and above, n (%)	468 (41.6%)	167 (41.8%)
75 y.o. and above, n (%)	207 (18.4%)	56 (14.0%)
Prevalence of AAA		
All, n (%)	28 (2.5%)	2 (0.5%)
55 y.o. and above, n (%)	26 (3.9%)	2 (0.6%)
65 y.o. and above, n (%)	24 (5.1%)	1 (0.6%)
75 y.o. and above, n (%)	15 (7.2%)	0(0%)

Data are presented as mean value ± standard deviation or n (%) of patients. AAA, abdominal aortic aneurysm; CKD, chronic kidney disease; DM, diabetes mellitus; y.o., years old.

Secondly, total 400 patients (male / female: 202 / 198) with DM were examined. Average age of DM group was 58.5 ± 15.9 years old. The number of patients 55 years old and above, 65 years old and above, and 75 years old and above were 271 (male / female: 137 / 134), 167 (male / female: 88 / 79) and 56 (male / female: 29 / 27), respectively (Table 4). The prevalence of AAA in the patients with DM was only 0.5% in all patients, 0.6% in patients 55 years old and above, 0.6% in patients 65 years old and above and 0% in patients 75 years old and above, respectively.

The prevalence of AAA in CKD group tended to increase along with age, while the prevalence of AAA in DM group did not. Indeed, the prevalence of AAA in the patients with DM was very low throughout the ages.

## Discussion

This study demonstrated for the first time that CKD was positively associated with the presence of AAA. In contrast, DM was negatively associated with the presence of AAA in Japanese population. Additionally, it is confirmed that the other risk factors for the presence of AAA in this study, such as HTN, IHD, DLP and smoking habit, accord consistently with those in western countries.

Previous screening studies reported that the estimated prevalence of AAA in western countries is 4 to 9% [1–6]. The prevalence in western countries is higher than that in Asian countries [28]. The Life Line Screening cohort study also showed a reduced risk for Asian-Americans (odds ratio 0.72) compared with Caucasian populations [11]. The incidence of AAA in Japanese was about 2.7% based on the data of Japanese autopsy [29]. The AAA Japan study, a large multicenter cohort study, showed that AAA is present in 4.1% of Japanese patients with HTN 60 year old and above [1]. In this study, the prevalence of AAA in patients with CKD 65 years old and above was 5.1%, relatively higher than previous two studies. Our study demonstrated that CKD is associated with the presence of AAA. There are a few studies that support our findings. A cohort study conducted at an integrated health care delivery system in northern California displayed that the renal insufficiency significantly associated with risk of AAA [18]. In a screening program at a regional Veterans Affairs health system study, Chun KC, et al. identified renal insufficiency as a risk factor. They found that eGFR < 60 mL/min/1.73m^2^ was significantly associated with AAA (36.7% vs. 24.3%; *P* < 0.001) [30]. A European group described that the stage of CKD revealed significant differences between AAA patients undergoing elective open repair surgery and controls [31], and the group also reported that 563 AAA patients prior to surgical repair contained 35.4% of stage III-V CKD [32]. However, these studies did not evaluate proteinuria, as an independent factor for AAA in their cohort. In another study, renal insufficiency significantly increased local levels of MMP-2 [33]. Both in the animal models and human studies, aortic aneurysm correlated with an increase in aortic MMPs levels such as MMP-2, -3, -9 and -12 [34–38]. In an animal model, Ziyi Liu, et al. reported an association between hyperhomocysteinemia (HHcy) and AAA (odds ratio, 7.39) in a meta-analysis, furthermore they observed HHcy aggravates AAA formation via activating adventitial fibroblast NADPH oxidase 4 in angiotensin II–infused male apolipoprotein E deficient mice. [39] Since CKD is well-known risk factor for atherosclerosis and CVD [13–16], it makes a great deal of sense that CKD is associated with AAA. In most cases, AAA is accompanied by HTN and atherosclerotic lesions. HTN and atherogenic condition often cause nephrosclerosis. Thus, it is likely that AAA is often associated with renal dysfunction, that is, CKD. Also, AAA usually presents at suprarenal, pararenal, or juxtarenal lesion, which is involved with or is located near the renal arteries. As a result, the blood flow to the renal artery becomes abnormal, and this blood flow carries cholesterol crystal and mural thrombus from the AAA wall to the renal artery, resulting in the failure of renal function. Therefore, it is likely that AAA is related with CKD. On the other hand, if Renin-Angiotensin-System (RAS) inhibitors are administered to the patients with AAA, these agents exert a protective role on the kidney, especially to reduce their urinary proteins and blood pressure, leading the improvement of CKD. In our study, as shown in Table 1, AAA cases had more prevalence of CKD although AAA cases took more RAS inhibitors than Non-AAA. Accordingly, these findings may emphasize the positive association between AAA and CKD.

DM is well-established risk factor for CVD, and is associated with increased long term mortality of arterial diseases [40–42]. However, a protective effect of DM on AAA was observed in several studies [4, 43–46]. A previous study revealed the inverse association between DM and aortic dilatation in 351 patients with advanced coronary artery disease in Japanese population [20]. Furthermore, a German study reported that wall thickness of AAA was increased in DM patients [47]. In the present study, we revealed that DM was negatively associated with AAA in Japanese population. Also, the prevalence of AAA in patients with DM 65 years old and above was 0.6%, clearly lower than the prevalence of AAA in general population of 2.7% [29]. A few previous studies suggested the potential mechanism of the protective effect of DM on AAA. Elevated glucose in patients with DM increases glycation of protein precursors resulting in increased formation of advanced glycation end products (AGEs) [22, 24]. AGE-mediated cross-links of collagen and elastin in aortic wall, reduces elasticity and increases the arterial stiffening and resistance to proteolysis by the mechanism reported that glycated matrix impairs MMP-1 synthesis and MMP-2 activation, and AGEs stimulate collagen synthesis via protein kinase C and TGF-*β*1 upregulation [23]. AGEs might increase smooth muscle cell proliferation and reduce the aneurysmal expansion and rupture [21, 22, 25, 26]. Furthermore, it is reported that patients with DM have reduced levels of MMP-2 and MMP-9, inhibiting the breakdown of the extracellular matrix proteins in the aortic wall [48, 49]. Further study is needed to elucidate the precise mechanism how DM prevents AAA.

As is the case in our study, the several risk factors for AAA overlapped with those for atherosclerosis. Indeed, atherosclerotic changes are frequently observed in aortic wall of AAA patients. The patients with AAA are often associated with multiple risk factors for atherosclerosis. The TromsøStudy, a population-based study of 6,386 men and women in Tromsø Norway, indicated that risk factors for atherosclerosis are associated with increased risk for AAA [5]. Rodin MB, et al. reported that the major risk factors for atherosclerosis, such as hyperlipidemia, HTN and smoking habit were positively and independently associated with AAA [50]. Several studies showed the positive correlation between CVD and AAA [18, 30, 51]. A cohort study in Northern California confirmed that major atherosclerotic risk factors including IHD and arteriosclerosis obliterans are related to AAA [18]. Our study also demonstrated that HTN and IHD were positively associated with AAA.

### Limitation

The present study had several limitations. First, some reports defined AAA as focal dilation of the abdominal aorta that are 50% greater than the proximal normal segment or that are ≥ 3.0 cm. However, we defined AAA as the maximum abdominal aortic diameter in minor axis ≥ 3.0 cm on CT according to ACC/AHA Guideline. Second, this study was retrospective case-control cross sectional study. Family history, socioeconomic status, histological and genetic information were not obtained enough to examine, because of lack of data in the physician’s charts. Third, there was a sampling bias in the present study, since we recruited the hospital-based population who received CT scanning as controls, in order to align the background of the patients. In addition, as previously described, we accordingly enrolled elderly patients. Therefore, each group had higher prevalence of DM than general population. Fourth, the definition of DM in this study was that patients were already diagnosed and using medical treatment or their HbA1c was 6.5% and above. In this retrospective study, there was few data that could be evaluated constant blood glucose levels. Since blood glucose data were not precisely assessable, we could not add these data to the analysis. In addition, we did not investigate the patients with diabetic nephropathy in detail because of the small number of participants. Fifth, this study included only the Japanese population. The differences among the races were not examined. Sixth, this study included only two centers’ patients and relatively small number of patients were examined.

## Conclusion

**Fig 1:**
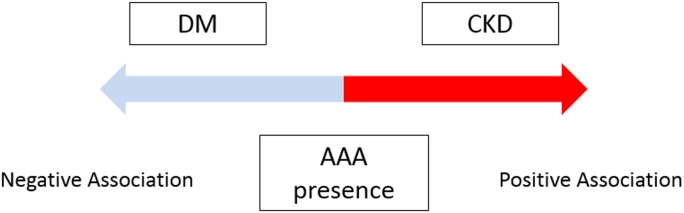
Schema of the relationship among CKD, DM and AAA. CKD is one of the positive determinants, meanwhile, DM is one of the negative determinants, for AAA presence.

We found that CKD is the independently associated with the presence of AAA, in contrast, DM is independently and negatively associated with the presence of AAA in Japanese population (Fig 1). The other risk factors, such as HTN and IHD, were confirmed as independently determinants for AAA. Our findings propose the idea that the screening examination by ultrasonography for CKD patients may detect AAA in earlier stage, then these patients can be properly treated to avoid the fatal events. Further large number, prospective studies and basic research are required to investigate the association and the underlying mechanisms of AAA with CKD and DM.

## Supporting Information

S1 Table Data of the detailed continuous variables. (DOCX)
