# Supplementary material for: Chronic Kidney Disease Is Positively and Diabetes Mellitus Is Negatively Associated with Abdominal Aortic Aneurysm
Source: PLoS One. 2016 Oct 20;11(10):e0164015. doi: 10.1371/journal.pone.0164015 (PMC5072712; doi:10.1371/journal.pone.0164015)
Supplement: S1 Table — (DOCX) [file pone.0164015.s001.docx]

**S1 Table. Data of the detailed continuous variables**

| AAA+ | | | | | | | |
| --- | --- | --- | --- | --- | --- | --- | --- |
|  | Age | Height | Weight | BMI | Cr | eGFR | HbA1c |
| n | 261 | 237 | 243 | 237 | 256 | 256 | 197 |
| Mean | 77.0 | 160.6 | 58.6 | 22.7 | 1.28 | 54.4 | 5.7 |
| SD | 8.3 | 8.8 | 11.8 | 3.8 | 1.19 | 21.2 | 0.6 |
| Median | 79.0 | 161.5 | 57.5 | 22.6 | 0.97 | 56.7 | 5.6 |
| 25% IQR | 72.0 | 155.3 | 51.1 | 20.3 | 0.80 | 41.1 | 5.3 |
| 75% IQR | 82.0 | 166.5 | 66.0 | 24.6 | 1.28 | 68.2 | 6.0 |
| Min | 44.0 | 131.5 | 31.2 | 13.3 | 0.40 | 3.8 | 4.2 |
| Max | 98.0 | 183.5 | 105.0 | 40.2 | 11.62 | 117.2 | 8.8 |

| AAA- | | | | | | | |
| --- | --- | --- | --- | --- | --- | --- | --- |
|  | Age | Height | Weight | BMI | Cr | eGFR | HbA1c |
| n | 261 | 238 | 245 | 237 | 260 | 260 | 242 |
| Mean | 77.0 | 159.5 | 56.3 | 22.1 | 1.18 | 61.4 | 5.9 |
| SD | 8.3 | 8.4 | 11.1 | 3.8 | 1.27 | 26.2 | 0.9 |
| Median | 79.0 | 160.0 | 55.4 | 22.1 | 0.87 | 62.3 | 5.7 |
| 25% IQR | 72.0 | 153.2 | 48.0 | 19.4 | 0.72 | 49.5 | 5.3 |
| 75% IQR | 83.0 | 165.0 | 63.2 | 24.1 | 1.07 | 72.8 | 6.2 |
| Min | 44.0 | 130.4 | 30.9 | 13.6 | 0.21 | 4.0 | 3.9 |
| Max | 98.0 | 182.0 | 98.0 | 38.8 | 11.30 | 159.8 | 9.9 |

AAA, abdominal aortic aneurysm; BMI, body mass index; Cr, creatinine; eGFR, estimated glomerular filtration rate; Hb, Hemoglobin; SD, standard deviation; IQR, interquartile range; Min, minimum; Max, maximum.

The other data are shown in the manuscript.
